# Analgesic Effect of Low Dose Subcutaneous Ketamine Administration Before and After Cesarean Section

**DOI:** 10.5812/ircmj.15506

**Published:** 2014-03-05

**Authors:** Kaveh Behaeen, Mansour Soltanzadeh, Sholeh Nesioonpour, Ahmad Ebadi, Alireza Olapour, Seyed Mohammad Mehdi Aslani

**Affiliations:** 1 Pain Research Center, Ahvaz Jundishapur University of Medical Sciences, Ahvaz, IR Iran; 2 Department of Anesthesiology, Ahvaz Jundishapur University of Medical Sciences, Ahvaz, IR Iran

**Keywords:** Ketamine, Caesarean Section, Pain, Postoperative, Diclofenac

## Abstract

**Background:**

Pain is considered as an importantissue after cesarean section. Multimodal approach to post cesarean pain management may not only enhance analgesia but also reduce side effects after the surgery.

**Objectives:**

This study was aimed to evaluate the clinical efficacy of subcutaneous injection of low dose ketamine at the incision site to reduce cesarean section pain.

**Patients and Methods:**

Sixty patients, aged between 18 and 25 years old, scheduled for elective cesarean section, were enrolled to this double-blind randomized controlled trial study. Patients were divided into three groups of 20 patients each group one (k-pre) received 0.5 mg/kg ketamine before skin incision and normal saline after skin closure, group two (k-post) received normal saline before skin incision and 0.5 mg/kg ketamine after skin closure and group three (C) received normal saline before skin incision and after skin closure; subcutaneously at the incision site. The first analgesic request, the amount of analgesic and the pain intensity were evaluated for 24 hours.

**Results:**

The first time analgesic requested was longer and the amount of analgesic used during the first 24 hours was significantly lower in groups K-pre and K-post compared with group C (P < 0.05). Pain intensity was significantly lower at 2, 4, 6 and 12 hours in groups K-pre and K-post compared with group C (P < 0.05). Nevertheless, pain intensity was not significantly different at 18 and 24 hours in group C (P > 0.05). The first requested time, total used amount of analgesicand pain intensity were not meaningfully different in K-pre and K-post groups (P > 0.05).

**Conclusions:**

Patients who were given ketamine before or after cesarean section subcutaneously at incision site had lower pain intensity and less analgesic consumption than patients who were given placebo.

## 1. Background

Pain - a sensational and emotional experience due to tissue injury - is one of the most important reasons for the patients’ maladies after surgery. Many studies have shown that the use of analgesic drugs reduces the postoperative complications, however in most cases the pain is not controlled adequately (1-3). Postoperative pain is also one of the most important concerns of women after cesarean section and it can hinder mother’s ability for desirable attention and the nutritional care of the newborn. Risk of thromboembolic disease, which increases during pregnancy, is likely to further exacerbate by immobility due to the pain during the puerperium. Chronic pain is one of the complications after cesarean section, which is reported for 12.3% of the cesarean sections. Severe acute postcesarean delivery pain is associated with persistent pain and postpartum depression for eight weeks after delivery (4, 5).

The studies have shown that preemptive pain control results in weakening afferent pain signals towards spinal cord, which appears to be far more effective than controlling the pain after its occurrence (6). There exists a basic hypothesis about pain transmission that is sensitization of spinal cord by activating glutamate and aspartate in N-Methyl-D-aspartate (NMDA) receptors (7). Recognition of NMDA receptors and the roles they play in reducing the patients’ pain causes a novel evolution in consumption of such receptor antagonists like ketamine (8-10). Using low-dose ketamine as the antagonist of NMDA receptors has been raised to not only alleviate the patients’ pain but also to lessen their needs for systemic opioid. This drug blocks NMDA; receptors on the postsynaptic membrane of posterior horn of spinal cord inhibits the pain transmission from pain pathway to the central nervous system and consequently diminish the pain, or in other words creates analgesia. Note worthily, new research have shown that ketamine has a local analgesic effect (11). Adding ketamine to local anesthetics or other analgesic drugs and their prescription around peripheral nerves or in spinal space has improved the analgesia and prolonged the duration of the reduced pain (12, 13). Moreover, local use of ketamine - aiming to affect peripheral receptors - can reduce the possible side effects such as psychological reactions and sedation (14, 15).

## 2. Objectives

This study aimed to evaluate the analgesic effect of subcutaneous low dosage of ketamine at incision site before and after cesarean section under spinal anesthesia.

## 3. Patients and Methods

This study was a double blind and prospective clinical trial that was conducted during the year 2013. The case and the control groups were all selected from patients being referred to Ahvaz Imam Khomeini Hospital in Iran. The approval was granted by Ethics Committee of Ahvaz Jundishapur University of Medical Sciences (ETH 641, 12/19/2012) and a written consent was taken from patients after providing them the detailed information of the steps, methods and drugs. The researcher was available there to answer the questions and the patients were allowed to leave the study whenever they decided. Sixty pregnant women aged between 18 to 25 years old with the inclusion criteria of first or second pregnancy and American Society of Anesthesiology class I (ASA I) who were candidates for elective cesarean section with Pfannenstiel incision were selected. The patients were divided into three groups of 20 persons on a random basis through a computer-generated list of random numbers. Patients had a known history of hypertension, hyperthyroidism, psychiatric disorders, chronic pain syndrome, allergy to ketamine, renal or hepatic insufficiency, seizure or intracranial hypertension and drug or alcohol abuse in the preceding six months. Notably, spinal anesthesia refusal was excluded. Electrocardiogram monitoring, pulse oximetry and blood pressure measurement were performed for the patients. All patients were hydrated with 10 mL/kg of Ringer’s solution. Then, spinal anesthesia was completed perform by 60 mg lidocaine 5% (Orion pharmacy, Finland) in the sitting position with mid line technique and needle number 25 gauge (Dr. J Co, China) at L3-L4 level, after aspiration of 0.2 cc of the cerebro spinal fluid. After ensuring the neuraxial blockade at T4 dermatome based on needle’s tip sensation, in the first group (K-pre), 0.5 mg/kg ketamine (Rolex Medica Co, Germany), reached volume of 10 cc by normal saline, was injected subcutaneously in the same volume and within equal intervals at the incision site before skin cut. Again, another 10 cc of normal saline was injected subcutaneously with equal intervals and volumes at the end of the surgery and after skin closure.

Given the second group (K-post), 10 cc of normal saline was injected subcutaneously before skin incision and 0.5 mg/kg ketamine with volumes of 10 cc administered at the incision site subcutaneously after skin closure. For the third group (C), 10 cc of normal saline was injected subcutaneously with the same intervals and volumes before skin incision and after skin closure. All the surgical incisions were started five minutes after the first prescription. It is worth mentioning that the gynecologists and the patients did not know about the group classifications. Same syringes 10 cc were used for all the cases and the drug was prepared by the anesthesiologist. Systolic and diastolic blood pressures, O_2_ saturation and heart rate of the patients were monitored after spinal anesthesia with five minutes intervals. The patients received 5 mg ephedrine on the condition of O_2 _% fall of systolic blood pressure. Moreover, they would also receive 0.5 mg atropine if the heart rate reached less than 60 bpm. The related dosages would be repeated if needed.

All the patients received six liters of oxygen per minute with a surgical mask. The systolic and diastolic blood pressure as well as heart rate of the patients were evaluated after sending them to recovery room. Patients were discharged from recovery room after the return of pain sensation level to T10 dermatome when they reacted to recognition of needle tip. After educating the patients, the intensity of the postoperative pain was surveyed at 2, 4, 6, 12, 18 and 24 hours after the beginning of anesthesia measured by V visual analogue scale (VAS) in which the minimum pain score is zero and the maximum is 10. Duration of the surgery was recorded as the first incision occurred until the last suture of the skin. The questionnaire was completed by the residents of anesthesiology who were uninformed of the drugs’ type. The patients received diclofenac if they had the VAS score of three or above (along with 75 mg intramuscular and then 100 mg suppository in case of need). The first analgesic requested from the beginning of the anesthesia and the total needed amount of analgesic were recorded. The side effects such as dizziness, allergic reactions, nausea, vomiting and hallucination were evaluated in the recovery room at 2, 4, 6, 8, 12, 18 and 24 hours after the commencement of anesthesia.

### 3.1. Statistical Analysis

Calculating the power of 95% and the confidence coefficient of 95%, the sample size was 16 in each group which were 48 samples in total by this formula: S_1_ = 0.7, S_2_ = 0.5, X_1_ = 2.3, X_2_ = 1.5. But then again, for greater certainty, 60 samples were selected, 20 samples each group. Normal distributed statistical data mean ± standard deviation (SD) and non-normal statistical data were reported based on median ± IQR. Comparing the studied groups was accomplished by one-way ANOVA statistical test, and Tukey’s post-hoc test were used after analyzing normal distributed data. Kruskal Wallis, Mann Witney U-test and Alfa correction were also employed after analyzing non normal data. Bonferroni test was used for repeated data measurement. P value < 0.05 was considered significant. Statistical analysis was performed using SPSS version 16.

## 4. Results

In this study, 60 patients were divided into three groups and there was no patient excluded: group received normal saline only (C), group with ketamine subcutaneously at incision site before surgery and skin incision (K-pre), and the group received ketamine subcutaneously at incision site after the surgery and skin closure (K-post). As shown in Table 1, the differences between the three groups were not substantial according to demographic characteristics (age, weight and height) and duration of surgery. Random assignment was performed accurately (P > 0.05). According to Table 2, the first analgesic request time after the surgery from the beginning of the anesthesia was significantly longer in K-pre and K-post groups compared to group C (P = 0.000). However, no significant difference was seen between the two groups receiving ketamine concerning the first analgesic request time (P = 0.809). the total amount of diclofenac used after the surgery was significantly lower in K-pre and K-post groups compared to group C (P = 0.005 and P = 0.001, respectively) (Table 3). Although, no significant difference existed in the total amount of analgesic used for the two groups receiving ketamine (P = 0.436).

**Table 1.** Demographic Characteristics and Duration of Surgery

	Group C	Group K-pre	Group K-post	P Value
**Age, y**	22.4 ± 1.77	22.7 ± 2.11	22.5 ± 2.5	0.951
**Weight, kg**	75.2 ± 3.82	76.6 ± 4.29	75.4 ± 4.1	0.777
**Height, cm**	158.7 ± 2	158.6 ± 2.87	157.3 ± 4.02	0.856
**Duration of surgery, min**	46.7 ± 3.4	47.7 ± 5.59	46.1 ± 3.95	0.719

**Table 2.** First Analgesic Request Time ^a^, ^b^

	Group C	Group K-pre	Group K-post
**First analgesic request time, min**	97.8 ± 6.59	202.40 ± 15.77	206.00 ± 14.49
**P value**	< 0.05	0.00	0.00

^a^ Data are presented in Mean ± SD.

^b^ Static test, one-way ANOVA, Tukey’s post-hoc test.

**Table 3.** Total Used Dosage of Analgesic ^a^, ^b^

	Group C	Group K-pre	Group K-post
**Total used dosage of analgesic, mg**	275 ± 00	175 ± 100	75 ± 100
**P value**	< 0.05	0.005	0.001

^a^ Data are presented in Median ± IQR.

^b^ Statics test: Kruskal Wallis, Mann Witney U-test, Alfa correction test.

**Figure 1.**
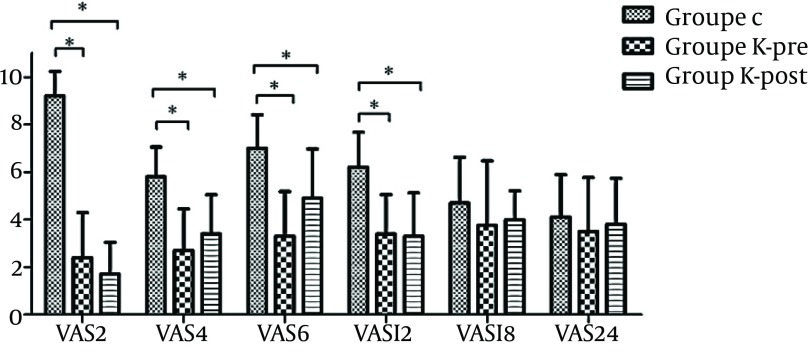
Mean and SD of Pain Intensity (VAS) Asterisk (*) show a significant difference between groups (P < 005).

As depicted in Figure 1, pain intensity after operation using VAS score in 2, 4, 6 and 12 hours after the beginning of anesthesia reduced significantly in K-pre and K-post groups compared to group C (P < 0.05). No significant difference was recorded in the postoperative pain intensity based on VAS score in 2, 4, 6 and 12 hours after the beginning of anesthesia in K-pre and K-post groups (P < 0.05). The mean pain intensity in the three groups did not show any significant difference at 18 and 24 hours after the beginning of anesthesia (P > 0.05). The Patients’ pain intensity based on VAS score was significantly higher in group C when compared two hours with four hours after the beginning of anesthesia (P = 0.000), while no notable difference was observed in K-pre and K-post groups (P = 0.07, P = 0.33, respectively). Last but not least, no side effects such as dizziness, allergic reactions, nausea, vomiting and hallucination were reported in any of the groups.

## 5. Discussion

The results of this study showed that the subcutaneous prescription of low dose ketamine (0.5 mg/kg) as antagonist of NMDA receptor before and after surgery can reduce the postoperative pain during the first 12 hours in comparison with receiving placebo. In addition, the subcutaneous consumption of ketamine before or after the surgery can not only delay the first analgesic request time but also reduce the total dosage of the analgesic compared with the control group. In this study, the drug types as well as the dosage of spinal anesthesia and local anesthetic were the same during all the stages; moreover, no opioid was used. Significant difference was seen in the patients’ pain intensity, revealing that the used amount of NSAIDs may be due to the subcutaneous prescription of ketamine in this study.

In a similar study, Honarmand et al. maintains that the subcutaneus or intravenous prescription of 0.5 mg/kg ketamine before the incision effectively reduces the postoperative pain as well as the amount of opioid used in appendectomy (16). Dal et al. also points out that the local intra articular use of ketamine with the dosage of 0.5 mg/kg can significantly decrease the VAS score amount in the patients undergoing arthroscopic surgery and improve knee function as well (17). Furthermore, Menkiti et al. states that intravenous ketamine with a dose of 0.15 mg/kg can considerably moderate the post-operative pain after cesarean section (18). Ketamine has a half-life of 15 minutes, hence it causes postoperative pain relief in earlier postoperative period and can be used as an additive analgesic for post-operative pain (16). Although the efficacy of ketamine administration before and after surgery has not been examined in these studies, Kwok et al. argued that the preincision administration of low dose intravenous ketamine (0.15 mg/kg) in gynecologic laparoscopic surgery was more effective concerning post-operative pain and first analgesic request time rather than post-surgical administration (19). The reason is that coupling ketamine with NMDA receptors may inhibit the activation of NMDA receptors induced by glutamate in principal afferent axons of the skin. As a result, it restrains afferent activation to communicate messages to spinal cord and suppresses sensitization of posterior horn (16).

In the conduced study, there was no substantial difference in the rate of the postoperative pain and the total used analgesic between the two groups receiving ketamine before and after surgery which was the same as the results of Menigaux et al.’s study (20) regarding the prescription of low dose ketamine for pain relief after anterior cruciate ligament repair. There could be two possible factors for not showing any difference in the two groups who received ketamine. First, the regional anesthesia like spinal or local anesthesia unlike the general anesthesia, inhibited the transmission of nociceptive nerve messages from the incision site to the spinal cord (21). Second, as the required time for subcutaneous ketamine to start having effect is between 5-15 minutes (16) according to time limitation in cesarean section after spinal anesthesia-the minimum needed time was taken into account for the starting point of the pharmacological effects. As Safavi et al. states higher dosages of subcutaneous ketamine up to 2 mg/kg reduce the post-operative pain significantly in the cholecystectomy; while, Honarmand et al. believes that doubling the local dosage of ketamine to 1 mg/kg intonsil surgery does not make difference in controlling the post-operative pain (22, 23). In this study, low dosage of ketamine was used to control the postoperative pain. Since, known psychomimetic complications of ketamine along with its analgesic effects with low plasma concentration of 100-200 ng/mL warned us in paying more attention to administering low dosages of ketamine when it comes to curing clinical pain (24).

In Kwok et al.’s study, no patient experienced problems caused by ketamine such as delusion, illusionand nightmare disorders due to the low dosage of intravenous ketamine (0.15 mg/kg) (19) similar with the results achieved by Honarmand et al. (16). The limitations of this study were the follow-up period spanned only during the first 24 hours in a way that the serum levels of ketamine and/or corticol after incisional injection were not measured, which could result in improving the accuracy of study. Cultural differences among the patients might have also influenced the results of the study. All in all it can be concluded that the subcutaneous prescription of low doses of ketamine before or after surgery is not only safe but also lessens the postoperative pain and consequently reduces the amount of analgesic used.
